# Supplementary material for: Magnetofermionic condensate in two dimensions
Source: Nat Commun. 2016 Nov 16;7:13499. doi: 10.1038/ncomms13499 (PMC5116097; doi:10.1038/ncomms13499)
Supplement: Supplementary Information — Supplementary Figures 1-3, Supplementary Note 1 and Supplementary References. [file ncomms13499-s1.pdf]

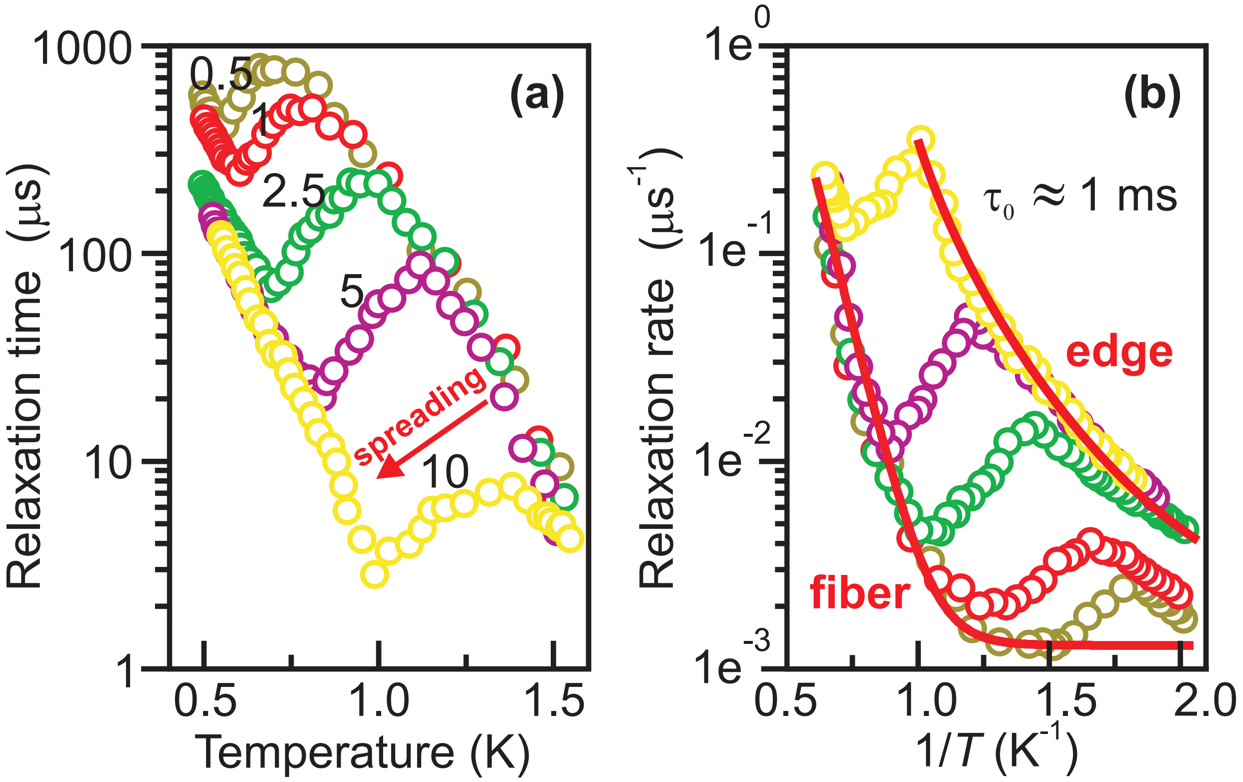

Supplementary Figure 1 | Pumping power effect on relaxation rate. Relaxation time **(a)** and corresponding relaxation rate **(b)** at different pumping levels in the range of 0.5-10 mW cm^{-2} for sample ♯2. The bottom red curve in **(b)** is the sum of two rates, the activated and the constant ones: $1/\tau = (1/\tau_1)exp^{-\Delta/k_{\rm B}T} + 1/\tau_0$, related to the finite lifetime of a single magnetoexciton at zero temperature with single magnetoexciton relaxation time $\tau_0 \approx 1$ ms, the singlet-triplet activation gap separating the "dark" magnetoexciton and magnetoplasmon energies Δ of 10 K, and magnetoexciton-magnetoplasmon conversion time $\tau_1 \approx 3$ ns. The upper red curve in **(b)** is an eye cursor indicating the relaxation rate for the magnetofermion condensate spread through the sample.

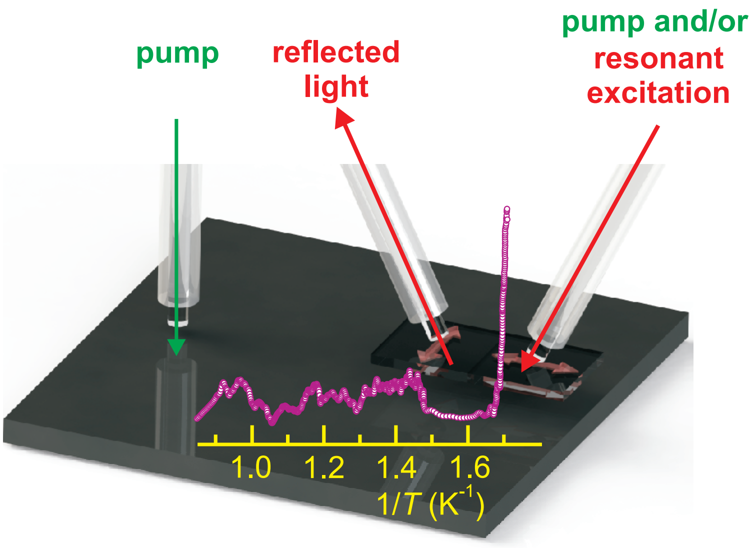

Supplementary Figure 2 | Experimental setup. The right part of the scheme illustrates the photoinduced resonant reflection (PRR) experiment. The left extra fiber is used for the PRR with spatial separation. The inset shows the temperature dependence of the PRR detected $\sim$ 2 mm away from the excitation spot.

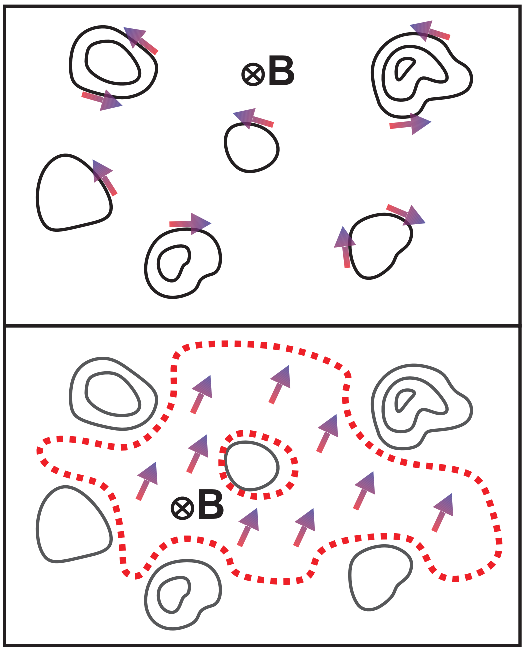

Supplementary Figure 3 | Condensation in pseudomomentum space. Incoherent (top) and coherent (bottom) magnetoexciton ensembles. The black curves indicate equipotential lines of the random potential. The arrows illustrate the magnetoexciton pseudomomenta. The area confined by red dashed line is a condensate cluster.

SUPPLEMENTARY NOTE 1 OPTICAL ABSORPTION IN INCOHERENT AND COHERENT STATES

The single magnetoexciton represents an asymptotically exact solution of the many-electron Schrödinger equation considered within the leading approximation in terms of the small parameter – the ratio of characteristic Coulomb energy $E_{\rm C}$ ($E_{\rm C}=\langle\varepsilon_{\rm q}\rangle\lesssim e^2/\kappa l_{\rm B}$) to cyclotron energy $\hbar\omega_{\rm c}^{\rm (e)}$. Concentration of magnetoexcitons achieved experimentally is $N/N_\Phi\simeq 0.1$ (N_Φ is the total number of states in the Landau level). We study the sharp increase of resonance light absorption (and corresponding resonance reflection) which is related to emergence of a coherent state within the magnetoexciton ensemble. The magnetoexciton ensemble is considered in a 'dilute limit', i.e. ignoring the inter magnetoexciton interaction.

The dependence of magnetoexciton energy on the pseudomomentum $\mathbf{q}$ was calculated by C. Kallin and B. Halperin [1]. It is determined by formula $E_{\mathbf{q}}=\delta+\varepsilon_{\rm q}$, where

$$\varepsilon_{\rm q}=\int_0^\infty ds\, e^{-s^2/2}\mathcal{F}_{\rm ee}(s)\left(1-\frac{s^2}{2}\right)[1-J_0(sq)] \tag{1}$$

(J_0 is the Bessel function, $\mathbf{q}$ is measured in $1/l_{\rm B}$ units), and $\delta\equiv E_{\mathbf{0}}$ is the $q=0$ shift including the cyclotron, Zeeman and Coulomb energies. $\mathcal{F}_{\rm ee}(q)=\frac{e^2}{\kappa l_{\rm B}}\iint dz_1dz_2e^{-q|z_1-z_2|/l_{\rm B}}|\chi_{\rm e}(z_1)|^2|\chi_{\rm e}(z_2)|^2$, where $\chi_{\rm e}(z)$ describes the electron size-quantized functions in the quantum well.

A. Consider an incoherent state:

$$|{\rm ini},N\rangle=\mathcal{Q}^\dagger_{\mathbf{q}_N}\mathcal{Q}^\dagger_{\mathbf{q}_{N-1}}...\mathcal{Q}^\dagger_{\mathbf{q}_1}|0\rangle, \tag{2}$$

where operator $\mathcal{Q}^\dagger_{\mathbf{q}}=N_\Phi^{-1/2}\sum_p e^{-iq_{ix}(p+q_{iy}/2)}b^\dagger_p a_{p+q_{iy}}$ creates a magnetoexciton with 2D pseudomomentum $\mathbf{q}$ (c.f. [2] and references therein); $|0\rangle$ to denote the $\nu=2$ 'vacuum'-ground state with a fully occupied zero Landau level; $a^\dagger_p$ is an operator creating an electron on the zero Landau level with a spin directed oppositely the magnetic field, and $b^\dagger_p$ creates an electron on the upper Landau level with a spin directed along the magnetic field). Considering the general case under condition $N\ll N_\Phi$ where all $\mathbf{q}$'s are assumed to be different, one finds the norm: $\langle N,{\rm ini}\,|\,{\rm ini},N\rangle=1+O(N/N_\Phi)$. The perturbation operator responsible for light absorption has the form $\hat{\mathcal{A}}=A\sum_p V^\dagger_p a^\dagger_p$ ($V^\dagger_p$ is describing a valence-band hole creation operator). The action of the $\hat{\mathcal{A}}$ operator on $|{\rm ini},N\rangle$ results in the $A\sum_i|f,\mathbf{q}_i\rangle$ combination of N final states:

$$|f,\mathbf{q}_i\rangle=-N_\Phi^{-1/2}\sum_p e^{-iq_{ix}(p+q_{iy}/2)}V^\dagger_p b^\dagger_{p+q_{iy}}\prod_{j\neq i}\mathcal{Q}^\dagger_{\mathbf{q}_j}|0\rangle. \tag{3}$$

If $N\ll N_\Phi$, then each state $|f,\mathbf{q}_i\rangle$ has a norm equal approximately to unity. Matrix element for transition to the final state $|f,\mathbf{q}_i\rangle$ is $|M_i|^2=\langle\mathbf{q}_i,f|\hat{\mathcal{A}}|{\rm ini},N\rangle^2\approx|A|^2$. The following calculation of the absorption rate represents a procedure of summation over all possible final states

$$\mathcal{R}_{\rm A}=(2\pi|A|^2/\hbar)\sum_i\delta(D_{\mathbf{q}_i}). \tag{4}$$

The used approach is single-excitonic, hence

$$D_{\mathbf{q}}=\hbar\omega+E_{\mathbf{q}}-E_{{\rm v-e},\mathbf{q}}, \tag{5}$$

where ω is the probing laser-beam frequency, $E_{\mathbf{q}}$ the total energy of the magnetoexciton $\mathcal{Q}^\dagger_{\mathbf{q}}|0\rangle$, and $E_{{\rm v-e},\mathbf{q}}$ the energy of creation of the valence-hole–conduction-electron exciton

$$|{\rm v-e},q\rangle=N_\Phi^{-1/2}\sum_p e^{-iq_{ix}(p+q_{iy}/2)}V^\dagger_p b^\dagger_{p+q_{iy}}|0\rangle\,. \tag{6}$$

The following study, i.e. summation of the δ-functions in Eq. (4), becomes impossible without a certain concretization of the initial state (2), representing the distribution of the $\mathbf{q}_i$-numbers over their possible values. This distribution is established and determined by two competing effects: by thermal diffusion related to interactions with phonons, and by drift motion, where the drift velocity of the magnetoexciton

$\partial E_{\mathbf{q}}/\partial\mathbf{q}$ is determined by two parameters, namely, pseudomomentum $\mathbf{q}$ and smooth random electric field $\vec{\mathscr{E}}=-\boldsymbol{\nabla}\varphi(\mathbf{r})$, $[\mathbf{r}=(x,y)]$.

Let us study a domain with a linear dimension smaller than the spatial dispersion parameter Λ of the smooth random potential φ (see Supplementary Fig.3) but still much larger than the magnetic length. (For definiteness let Λ be the correlation length of φ with the mean value of the potential $\overline{\varphi}\equiv 0$.) Within this domain we use a gradient approximation considering field $\varphi(\mathbf{R})$ as well as gradients $\boldsymbol{\nabla}_R\varphi$ and $\boldsymbol{\nabla}_R\mathscr{E}$, and coordinate $\mathbf{R}$ as parameters inherent to the domain (for example, $\mathbf{R}$ to denote the domain center.) The electrostatic term in the Hamiltonian is equal to $\hat{\varphi}=\vec{\mathscr{E}}(\mathbf{R})\sum_i\mathbf{r}_i$ (here summation is over all electrons within the domain), and when choosing $\vec{\mathscr{E}}\parallel\hat{x}$, then in terms of the secondary quantization it is presented as

$$\hat{\varphi}=\mathscr{E}l_{\rm B}\left[\sum_{\sigma=\uparrow,\downarrow}(\hat{K}_\sigma^\dagger+\hat{K}_\sigma)-\hat{P}_y\right] \tag{7}$$

($\mathscr{E}$ denoting $|\vec{\mathscr{E}}|$), where

$$\hat{P}_y=\sum_{n,p,\sigma=\uparrow/\downarrow}p\,c_{np\,\sigma}^\dagger c_{np\,\sigma} \tag{8}$$

is analog of the y-component of the Gor'kov-Dzyaloshinsky operator $\hat{P}$ [3] rewritten in the 'generalized' form valid for a purely electronic system (see Ref. [4]; in particular, $\hat{P}\mathcal{Q}_{\mathbf{q}}^\dagger|0\rangle=\mathbf{q}\mathcal{Q}_{\mathbf{q}}^\dagger|0\rangle$). The operator

$$\hat{K}_\sigma^\dagger=\sum_{n,p}\sqrt{\frac{n+1}{2}}\,c_{n+1p\sigma}^\dagger c_{np\,\sigma} \tag{9}$$

is the Landau-level 'raising' operator; $c_{np\sigma}^\dagger$ is the creation operator for the n-th Landau level, e.g., $a_p\equiv c_{0p\downarrow}$ and $b_p\equiv c_{1p\uparrow}$. Now we can obtain the contribution of electrostatic term (7) to magnetoexciton energy $E_{\mathbf{q}}$. The first order correction is $\langle 0|\mathcal{Q}_{\mathbf{q}}|\hat{\varphi}|\mathcal{Q}_{\mathbf{q}}^\dagger|0\rangle\equiv -l_{\rm B}\vec{\mathscr{E}}\times\langle 0|\mathcal{Q}_{\mathbf{q}}|\hat{\mathbf{P}}^\dagger|\mathcal{Q}_{\mathbf{q}}^\dagger|0\rangle=l_{\rm B}(\mathbf{q}\times\vec{\mathscr{E}})_z$. In principle, the second order correction should be determined by the K-terms of operator (7). By so calculating attention should be given to the fact that the energy of the $\mathcal{Q}_{\mathbf{q}}^\dagger|0\rangle$ state must be counted off the ground state energy. However, the latter for its part also includes second order electrostatic correction, and besides, both corrections turn out to be equal. Thus, the difference vanishes and the total correction is reduced to a null result (This feature is due to the symmetry of the purely electronic system considered. Formally, as applied to the case of a common two-particle magnetoexciton [3], this symmetry would correspond to electron and hole masses satisfying the $m_{\rm e}=-m_{\rm h}$ equivalence.) .

Now we can write out the relevant energy of the magnetoexciton within the domain in the vicinity of the roton minimum. With an accuracy up to a constant independent of $\mathbf{q}$ and $\mathbf{R}$, the relevant part of the energy is

$$\mathcal{E}(\mathbf{q},\mathbf{R})=\alpha(q-q_{01})^2+l_{\rm B}(\mathbf{q}\times\vec{\mathscr{E}})_z\,, \tag{10}$$

where parameters α and q_{01} are positive. Knowing the growth parameters of the sample and using (1) and the local-density approximation [5, 6] α and q_{01} are easily calculated for the particular electron system in question $\alpha\simeq 0.4\,$meV and $q_{01}\simeq 0.85$. $\mathcal{E}$ takes on a local minimum value at $\mathbf{q}=\mathbf{q}_{\rm m}(\mathbf{R})=-\vec{\mathscr{E}}\times\hat{z}\,(l_{\rm B}/2\alpha+q_{01}/\mathscr{E})$ which is the root of equation $\partial\mathcal{E}/\partial\mathbf{q}=0$. The local minimum corresponds to zero velocity and, hence, to the zero drift velocity of the excited electron and the vacancy in the spin-up sublevel of the zero Landau level composing the magnetoexciton. Physically it means vanishing of the total electric field that acts on each quasiparticle since the dipole moment, $\mathbf{d}_{\rm m}=l_{\rm B}\hat{z}\times\mathbf{q}_{\rm m}$, induces a field preventing occurrence of an external one. It is natural to consider the initial metastable state corresponding to this minimum. Due to the fast cool-down and diffusion processes compared to the magnetoexciton lifetime, the magnetoexciton gets 'stuck' in the smooth random potential with energy $\delta'+\mathcal{E}_{\rm m}(\mathbf{q}_{\rm m},\mathbf{R})$, where

$$\mathcal{E}_{\rm m}(\mathbf{R})=-l_{\rm B}q_{01}\mathscr{E}-(\mathscr{E}l_{\rm B})^2/4\alpha. \tag{11}$$

(δ' is the constant independent of $\mathbf{R}$; c.f. the study of spin-exciton kinetics in Refs. [7, 8]). Thus, the system is a frozen chaotic state held by the smooth random potential (see the Supplementary Fig.3).

The final state $|{\rm v}-{\rm e},\mathbf{q}_{\rm m}\rangle$ emerging within the domain as a result of photon absorption is explicitly defined. It represents a 'common' 2D-exciton studied, for instance, in Ref. [9], considered, however, against

the background of the zero Landau level completely occupied by conduction-band electrons. This background is a rigid system whose state, if calculated to the first order in the Coulomb interaction, is not changed even in the presence of the magnetoexiton. The occupied electronic Landau level for its part does not influence the q-dispersion of the energy $E_{\mathrm{v-e},\mathbf{q}}$ studied within the same approximation. However, allowance should be made for the fact that the corresponding effective formfactor $\mathcal{F}_{\mathrm{ve}}(q)$ in the dispersion equation similar to Eq. (1) is still larger than $\mathcal{F}_{\mathrm{ee}}(q)$. (It occurs owing to the greater "compactness" of heavy-hole function $|\chi_{\mathrm{v}}(z)|^2$ compared to electron function $|\chi_{\mathrm{e}}(z)|^2$.) The dependence of the energy on the external random field can be studied in the way described above with the only change from field operator $\hat{\varphi}$ to operator $\hat{\varphi} - \vec{\mathscr{E}}(\mathbf{R})\,\mathbf{r}_{\mathrm{h}}$, where $\mathbf{r}_{\mathrm{h}}$ is the position of the valence hole within the domain. The first order correction in electric field $\mathscr{E}$ is the same as for the electronic magnetoexciton. Meanwhile the second order correction does not vanish. Indeed, first, the $\sim \mathscr{E}^2$ corrections to energies of purely electronic states $b_p^\dagger|0\rangle$ and $|0\rangle$ determined by the Eq. (9) operators, do not compensate each other. Second, the $\sim \mathscr{E}^2$ correction to state $V_p^\dagger|0\rangle$ should also be taken into account. As a result, the total correction is the same as that found by L.P. Gor'kov and I.E. Dzyaloshinsky [3]. Finally, the relevant part of $E_{\mathrm{v-e},\mathbf{q}_{\mathrm{m}}}$ energy is

$$\mathcal{E}_{\mathrm{v-e}}(\mathbf{q}_{\mathrm{m}},\mathbf{R}) = \beta(q_{\mathrm{m}} - q_{02})^2 + l_{\mathrm{B}}(\mathbf{q}_{\mathrm{m}}\times\vec{\mathscr{E}})_z - \frac{\mathscr{E}^2 l_{\mathrm{B}}^2}{2\hbar}\left(\frac{1}{\omega_{\mathrm{c}}^{(\mathrm{e})}} + \frac{1}{\omega_{\mathrm{c}}^{(\mathrm{h})}}\right), \tag{12}$$

where $\beta \simeq 0.5$ meV, $q_{02} \sim 0.9$ are calculated similar to α and q_{01}; $\omega_{\mathrm{c}}^{(\mathrm{e})}$ and $\omega_{\mathrm{c}}^{(\mathrm{h})}$ are the cyclotron frequencies in the conduction and valence bands, respectively. The last term in Eq. (12) is definitely small and, within the framework of the absorption mechanism studied here, it has to be taken into account only in a symmetric case when $\alpha \approx \beta$ and $q_{01} \approx q_{02}$. This term also becomes essential in section **B** below.

Domains participating in summation (5) must satisfy two conditions: (i) they contain a magnetoexciton and (ii) correspond to a vanishing argument of the δ-function. Therefore, up to a constant C independent of the coordinate, we come to the equation $\hbar\omega - \mathrm{C} = \mathcal{E}_{\mathrm{v-e}}(\mathbf{q}_{\mathrm{m}},\mathbf{R}) - \mathcal{E}_{\mathrm{m}}(\mathbf{R})$ for $\mathbf{R}$. This, by substituting expressions (11) and (12), can be rewritten as

$$F(\mathbf{R}) = \hbar\omega - \mathit{const}\,, \tag{13}$$

where

$$F(\mathbf{R}) = (\beta/\alpha) l_{\mathrm{B}}(q_{01} - q_{02})\mathscr{E} + (\beta - \alpha)(\mathscr{E} l_{\mathrm{B}})^2/4\alpha^2\,, \tag{14}$$

and *const* is a combination of the forbidden gap, the Coulomb, Zeeman, and cyclotron shifts relevant to the case (the $\propto \mathscr{E}^2/\omega_{\mathrm{c}}^{(\mathrm{e,h})}$ terms are ignored). Under the experimental conditions the frequency ω of the probing laser beam is appropriately tuned in order to ensure a maximum signal, i.e. to provide the maximum number of domains participating in the sum (4). The solution of Eq. (13) determines a certain line in the (X,Y) plane. We introduce local orthogonal coordinates s and u, where s is the length along the line, and unit vector $\hat{\mathbf{u}}$ is parallel to $\boldsymbol{\nabla}F$. In the general case of smooth and random field $F(\mathbf{R})$ (these features are related to smoothness and randomness of $\mathscr{E}$) the line represents a closed non-self-intersecting curve (loop) with a length determined by the inhomogeneity characteristic Λ and frequency ω. First, consider a certain domain $du\times ds$ belonging to the curve. If the distribution of magnetoexcitons over area $S = 2\pi l_{\mathrm{B}}^2 N_\Phi$ of the spot created by the pumping laser is equiprobable, then the probability to find out the magnetoexciton within the domain is $(N/S)duds$ and the contribution of the domain to the sum in Eq. (4) is $(N/S)ds\int\delta(|\boldsymbol{\nabla}F|u)du = Nds/S|\boldsymbol{\nabla}F|$. Summating over all such domains we estimate the contribution of a single loop to sum (4) as $N\mathscr{L}_\omega/S\overline{|\boldsymbol{\nabla}F|}$, where $\mathscr{L}_\omega$ is the loop length and $\overline{|\boldsymbol{\nabla}F|}$ is the mean value along the loop. It is natural to assume that at frequency ω corresponding to the maximum of the absorption signal the most contribution to the (4) signal is provided by 'standard' loops. For those we estimate $\mathscr{L}_\omega \sim \pi\Lambda'$ where Λ' is the linear characteristic of the inhomogeneity for the $\mathscr{E} = |\boldsymbol{\nabla}\varphi|$ field, and, hence, $\Lambda' \sim \Lambda$. A more delicate estimate shows that $\Lambda' = k\Lambda$ where $k < 1$. Indeed, for example, if it is assumed that $\mathscr{E} \simeq \sqrt{\overline{(\boldsymbol{\nabla}\varphi)^2}}$ and φ is a Gaussian random field, then $\overline{(\boldsymbol{\nabla}\varphi)^2} = 2(\Delta/\Lambda)^2$ [Δ describing the potential amplitude $\left(\overline{\varphi^2}\right)^{1/2}$]. Therefore, $\mathscr{E} \simeq \sqrt{2}\Delta/\Lambda$, and $k \simeq 1/\sqrt{2}$ in this case. By analogy we find $\overline{|\boldsymbol{\nabla}\mathscr{E}|} \simeq \Delta/\Lambda'^2$ and $\overline{|\boldsymbol{\nabla}\mathscr{E}^2|} \simeq \Delta^2/\Lambda'^3$. These estimates should be substituted into $\overline{|\boldsymbol{\nabla}F|} \sim (\beta/\alpha)l_{\mathrm{B}}|q_{01} - q_{02}|\overline{|\boldsymbol{\nabla}\mathscr{E}|} + (\beta - \alpha)\overline{|\boldsymbol{\nabla}\mathscr{E}^2|}l_{\mathrm{B}}^2/4\alpha^2$. The last step to perform the summation in Eq. (4) is calculation of the total number of standard loops corresponding to frequency ω. Intuitively this number is $\gamma S/(\pi\Lambda'^2/4)$ with factor $\gamma \simeq 1/4$ for standard loops. Multiplying it by the found above contribution of one standard loop, we obtain an estimate of absorption rate $\mathcal{R}_{\mathrm{A}} = f_{\mathrm{A}}N$, where f_{A} is the "oscillator strength":

$$f_{\mathrm{A}} = 2\pi|A|^2/\hbar\Phi\Delta\,. \tag{15}$$

In this formula

$$\Phi(B,\Lambda,\Delta)\sim|q_{01}-q_{02}|\frac{\sqrt{2}\beta l_{\rm B}}{\alpha\Lambda}+(\beta/\alpha-1)\frac{\Delta l_{\rm B}^2}{2\alpha\Lambda^2}\,. \tag{16}$$

Since α, β and $l_{\rm B}$ are inversely proportional to the square root of magnetic field B, the oscillator strength (15) should grow with growing B.

B. In this section we demonstrate how the light absorption rate can be estimated for the coherent state, where a considerable number of magnetoexcitons belongs to the same state. We consider a cluster with area $\mathcal{L}\times\mathcal{L}$, so that $N_\Phi=\mathcal{L}^2/2\pi l_{\rm B}^2$ now. Instead of (2), one has

$$|N\rangle=(\mathcal{Q}_{\mathbf{q}}^\dagger)^N|0\rangle. \tag{17}$$

The energy $E_{N\mathbf{q}}$ of this state does not depend on spatial fluctuations of the random electrostatic field if $\mathcal{L}\gg\Lambda$ (see Supplementary Fig.3). Indeed, at constant $\mathbf{q}$ summation of electrostatic contributions $l_{\rm B}(\mathbf{q}\times\hat{z})\boldsymbol{\nabla}\varphi(\mathbf{r})$ over the cluster area is reduced to integration $\propto\int d\mathbf{r}\boldsymbol{\nabla}\varphi(\mathbf{r})$ and thereby yields a zero result. The norm of (17) is calculated in the same way as it was done earlier in the case of $\mathbf{q}\equiv 0$. (Norm R_N was first calculated for the $\mathbf{q}=0$ case in [10]. The calculation was based on certain commutation rules for $\mathcal{Q}_{\mathbf{0}}^\dagger$-exciton operators (see therein and also in [11]). It can also be performed in a similar way based on commutation rules for the $\mathcal{Q}_{\mathbf{q}}^\dagger$ operators at arbitrary $\mathbf{q}$ (see, e.g., [12]) and results in the same formula.) The result, $R_N=\langle N|N\rangle=N!N_\Phi!/N_\Phi^N(N_\Phi-N)!$, does not depend on $\mathbf{q}$. So, the normalized initial state is $|N\rangle R_N^{-1/2}$. Operator $\hat{\mathcal{A}}$ is represented as a sum of p-components, $\sum_p\hat{A}_p$, where $\hat{A}_p=AV_p^\dagger a_p^\dagger$. The action of $\hat{A}_p$ on the normalized initial state results in a single state with fixed $\mathbf{q}$: $|p,N{-}1\rangle=-Ae^{-iq_x(p+q_y/2)}N(N_\Phi R_N)^{-1/2}V_p^\dagger b_{p+q_y}^\dagger|N{-}1\rangle$. The $|p,N-1\rangle$ states at different p are orthogonal. The norm is also independent of p and calculated exactly: $|M_{Np}|^2=\langle N{-}1,p\,|\,p,N{-}1\rangle=|A|^2N/N_\Phi$. Thus, we find the transition matrix element squared for operator $\hat{\mathcal{A}}$ as $|M_N|^2=\sum_p|M_{Np}|^2=N|A|^2$ being by factor N larger than $|M_i|^2$ found in the previous section.

Our task is calculation of absorption rate $\mathcal{R}_{\rm B}=2\pi(|M_{Np}|^2/\hbar)\sum_p\delta(\hbar\omega+E_{N\mathbf{q}}-E_{N-1\,\mathbf{q}}-E_{\text{v}-\text{e},\mathbf{q}})$. We again study a small domain determined by position $\mathbf{R}$ and change from summation over p to integration in real space $\int\!...dudv/2\pi l_{\rm B}^2$ within the domain and with the following summation over domains. Considering the difference $E_{N\mathbf{q}}-E_{N-1\,\mathbf{q}}-\mathcal{E}_{\text{v}-\text{e}}(\mathbf{q},\mathbf{R})$ within the domain [see Eq. (12) for $\mathcal{E}_{\text{v}-\text{e}}$] we conclude that first-order electrostatic terms $l_{\rm B}(\mathbf{q}\times\hat{z})\boldsymbol{\nabla}\varphi(\mathbf{R})$ again are cancelled in the initial and final states and do not enter the difference. Now the electrostatic contribution to the argument of the δ-function is related to the $\propto\mathscr{E}^2$ terms in Eq. (12). The situation differs from the previous one in replacement of field $F(\mathbf{R})$ with field $-\mathscr{E}^2(\mathbf{R})(1/\omega_{\rm c}^{(\rm e)}+1/\omega_{\rm c}^{(\rm h)})l_{\rm B}^2/2\hbar$. We choose unit vector $\hat{\mathbf{u}}$ directed along gradient $\boldsymbol{\nabla}\mathscr{E}^2(\mathbf{R})$. As a result, estimating again $|\boldsymbol{\nabla}\mathscr{E}^2|\sim\Delta^2/\Lambda'^3$, we find: first, the contribution of one 'standard' loop to the absorption rate; and, finally, multiplying by the number of standard loops within the cluster $\sim\mathcal{L}^2/\pi\Lambda'^2$ (assuming $\mathcal{L}\gg\Lambda$), the contribution of the cluster to the absorption rate $\mathcal{R}_{\rm B}=f_{\rm B}N$. Here the oscillator strength is

$$f_{\rm B}\simeq|A|^2\pi\omega_{\rm c}^{(\rm h)}(\Lambda/l_{\rm B}\Delta)^2 \tag{18}$$

(it is taken into account that $\omega_{\rm c}^{(\rm h)}<\omega_{\rm c}^{(\rm e)}$). Thus, the enhancement of the absorption/reflection signal due to forming the coherent state is $f_{\rm B}/f_{\rm A}$. Since the first term in Eq. (16) is much larger than the second:

$$f_{\rm B}/f_{\rm A}\simeq\frac{\hbar\omega_{\rm c}^{(\rm h)}\beta\Lambda|q_{01}-q_{02}|}{\sqrt{2}\alpha l_{\rm B}\Delta}\,. \tag{19}$$

Taking Δ as the bandwidth of the absorption band of 0.15 meV and measuring directly $\hbar\omega_{\rm c}^{(\rm h)}\approx 2.4$ meV one is left with a single unknown quantity Λ. Supposing as the lowest limit of the presented theory $\Lambda\simeq 10l_{\rm B}$ one immediately obtains the estimate $f_{\rm B}/f_{\rm A}\simeq 7$ which is in reasonable agreement with the experimental results $f_{\rm B}/f_{\rm A}\simeq 10$ (see Fig.2 in the main text).

SUPPLEMENTARY REFERENCES

[1] Kallin, C. & Halperin, B. I. Excitations from a filled Landau level in the two-dimensional electron gas. *Phys. Rev. B* **30,** 5655-5668 (1984).

[2] Dickmann, S. Extremely slow spin relaxation in a spin-unpolarized quantum Hall system. *Phys. Rev. Lett.* **110,** 166801 (2013).

[3] Gor'kov, L. P. & Dzyaloshinskii, I. E. Contribution to the theory of the Mott exciton in a strong magnetic field. *Zh. Eksp. Teor. Fiz.* **53,** 717-722, (1967) [*Sov. Phys. JETP* **26,** 449-451 (1968)].

[4] Dickmann, S. Goldstone-Mode Relaxation in a Quantized Hall Ferromagnet in the Presence of Smooth Random Potential. *arXiv:cond-mat/0312427* (see Ref. [16] therein).

[5] Hohenberg, P. & Kohn, W. Inhomogeneous Electron Gas. *Phys. Rev.* **136,** B864-B871 (1964).

[6] Kohn W. & Sham, L. J. Self-Consistent Equations Including Exchange and Correlation Effects. *Phys. Rev.* **140,** A1133-A1138 (1965).

[7] Zhuravlev, A. S., Dickmann, S., Kulik, L. V., & Kukushkin, I. V. Slow spin relaxation in a quantum Hall ferromagnet state. *Phys. Rev. B* **89,** 161301(R) (2014).

[8] Kulik L. V., *et al.* Super-long life time for 2D cyclotron spin-flip excitons. *Sci. Rep.* **5,** 10354 (2015).

[9] Lerner, I. V. & Lozovik, Yu. E. Mott exciton in a quasi-two-dimensional semiconductor in a strong magnetic field. *Zh. Eksp. Teor. Fiz.* **78,** 1167-1175, (1978) [*Sov. Phys. JETP* **51,** 588-592 (1980)].

[10] Dzyubenko, A. B. & Lozovik, Yu. E. Symmetry of Hamiltonians of quantum two-component systems: condensate of composite particles as an exact eigenstate. *J. Phys. A: Mat. Gen.* **24**, 415-424 (1991).

[11] Dickmann, S. M. & Iordanskii, S. V. Spin relaxation under conditions of the quantum Hall effect with odd filling. *Pis'ma Zh. Eksp. Teor. Fiz.* **63,** 43-48, (1996) [*Sov. Phys. JETP Letters* **63,** 50-55 (1996)].

[12] Dickmann, S. M. & Iordanskii, S. V. Spin relaxation of two-dimensional electrons with an odd Landau-level filling factor in a strong magnetic field. *Zh. Eksp. Teor. Fiz.* **110,** 238-269, (1996) [*Sov. Phys. JETP* **83,**, 128-145 (1996)].
